# Titanium Dioxide Photocleans Polluted Air

**DOI:** 10.1289/ehp.120-a229

**Published:** 2012-06-01

**Authors:** Adrian Burton

**Affiliations:** Adrian Burton is a biologist living in Spain who also writes regularly for *The Lancet Oncology*, *The Lancet Neurology*, and *Frontiers in Ecology and the Environment*.

Could *titanium-clean air* be how advertisers one day try to sell us the virtues of paint or even clothing that reduces the pollution caused by automobiles?

The technology behind such products involves the well-known ability of titanium dioxide (TiO_2_)to photocatalytically split water to form hydroxyl and peroxyl radicals,^1^ a reaction that has already been harnessed to make self-cleaning windows.^2^ Pacific Paints (Boysen) Philippines, Inc., is now promoting a paint it claims cleans air of automobile-produced nitrogen oxides (NO_X_)—known respiratory irritants and precursor molecules of ground-level ozone.^3^ In the presence of sunlight, TiO_2_ nanoparticles in the paint form hydroxyl and peroxyl radicals, which then react with NO_X_ in the air to produce nitric acid. This reacts with calcium carbonate in the paint matrix to produce minute quantities of calcium nitrate, water, and carbon dioxide (CO_2_). Another company, Alcoa, is marketing a coating for aluminum building panels that relies on the same kind of photochemistry,^4^ while Boral Roofing is using it to make pollution-reducing roof tiles.^5^

Cristal Global produces the CristalACTiV™ TiO_2_ nanoparticles currently used by Pacific Paints in their Boysen KNOxOUT™ paint. In one trial in Manila’s Guadalupe train station, Cristal Global painted 4,100 m^2^ of exterior wall and found the paint removed about 26 g of NO_X_ per 100 m^2^ of painted surface. The company claims each painted square meter could remove 80 g of NO_X_ per year. In another trial that ran over four years, a 135-m^2^ wall in London was treated with another Cristal product and the NO_X_ levels in its vicinity measured. Compared with control areas in other parts of the city, the company reported reductions as great as 60% for the NO_X_ species nitric oxide and 20% for the species nitrogen dioxide. The photocatalytic paint is even reported to work in the low-light environment of multistory parking lots, capturing 2.2 g of NO_X_ per square meter per year.^6^

Based on trial data, Cristal Global claims that a 100-m^2^ surface painted with KNOxOUT could, in the course of a year, remove the NO_X_ equivalent to that produced by a car driven more than 130 km.^6^ It is, of course, hard to predict exactly how much NO_X_ might actually be removed, given the difficulty of controlling city environments in outdoor trials.

“It is clear that these paints work,” says Sixto Malato, head of the Research Unit at Spain’s Plataforma Solar de Almería, an expert in photochemistry with no connection to the company. “But it’s also true that these photocatalytic materials deteriorate quite easily. How long they will stay functional is therefore unclear. I don’t know of any studies that guarantee durability for, say, five to ten years, the interval at which many walls, buildings, and garages might normally be painted. We need to undertake long-term durability studies in very controlled environments that might allow some official certificate of quality to be issued.”

**Figure:**
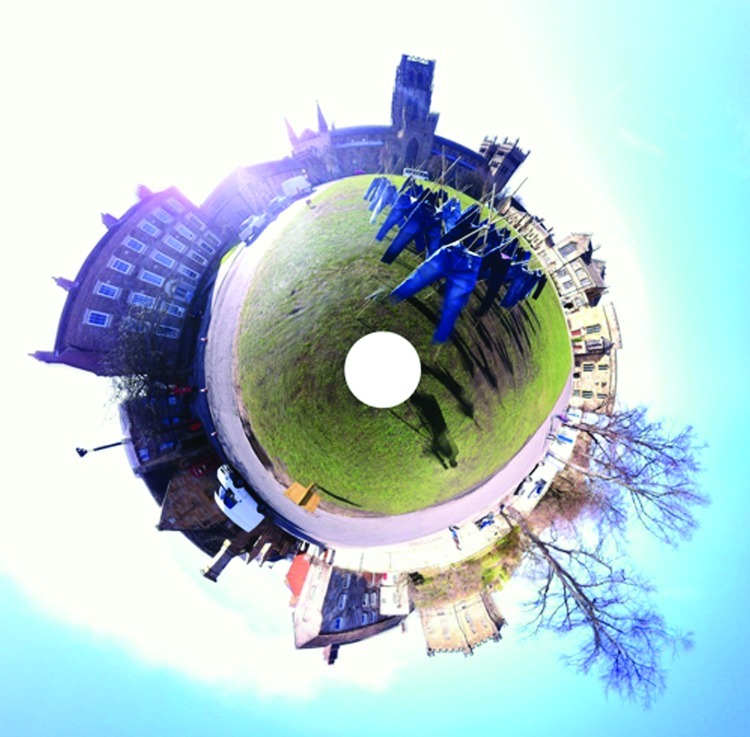
An exhibit of “catalytic jeans” is part of the Newcastle (U.K.) Science Festival 2012. ‘Field of Jeans’ Stereographic, Durham by DED http://www.dedass.com

Brian Pickett, business director for performance chemicals business at Cristal Global, says the paint may cost twice the normal price. “However, the major cost associated with applying [any] paint is in the labor, which does not change,” he says. “So the overall impact is about ten percent more than the application of a nonphotocatalytic paint.”

Meanwhile, researchers in the United Kingdom are making use of the same reactions to create NO_X_ -removing “catalytic clothing” in which TiO_2_ nanoparticles and calcium carbonate are added to fabrics, sprayed onto clothes, or applied during washing.^7^ “The method of delivery we are pursuing is via a laundry product,” explains Helen Storey, a professor of fashion and science at the London College of Fashion. “In this sense, any clothing that can be washed . . . would be able to carry the catalyst.”

Someone wearing all catalytic clothing all day could potentially remove 6 g of NO_X_ per day, or 2 kg a year, calculates Anthony Ryan, pro-vice-chancellor for the Faculty of Science at the University of Sheffield, who is involved in the project. He estimates four “catalyzed” people could take out the NO_X_ pollution from one European car producing 8 kg per year over 15,000 km. Under European clean-air rules enacted in 2008,^8^ the city of Sheffield must reduce its NO_X_ production by some 10% by 2015, from 9,000 to 8,000 metric tons per year. For that, “we’d need half a million people to be catalyzed—about half the population,” says Ryan.

The CO_2_ and nitrate products of NO_X_ degradation are, however, themselves pollutants. CO_2_ is a greenhouse gas, and nitrates pollute waterways, contributing to eutrophication. Excessive concen-trations of nitrates in drinking water cause the blood disorder methemoglobinemia and may also contribute to the risk of thyroid problems, adverse birth outcomes, and cancer.^9^

So are we not simply swapping one pollution problem for another? Ryan says NO_X_ is much more—and more immediately—harmful than these end products, but Malato believes we need to know more about the titanium dioxide lost to the wastewater system during washing, which might eventually enter rivers. “If activated by light, it would produce radicals that might harm aquatic organisms,” he says. Malato also expresses concern that the TiO_2_ nanoparticles used in these products might behave differently than conventional TiO_2_ in biological systems, and he warns that studies should be performed to detemine whether they present any additional health hazard.

Could innovative applications like these really mop up some of the harmful compounds we spill into the air? “Maybe some,” says Felix López, a professor of research with the Spanish National Research Council in Madrid. “But the *real* solution, of course, is not to pollute in the first place.”
